# The relationship between radial artery spasm and adropin levels in patients undergoing transradial coronary angiography

**DOI:** 10.34172/jcvtr.2022.15

**Published:** 2022-06-14

**Authors:** Fuat Bice, Mehmet Eyuboglu, Zeliha Cansel Ozmen, Baris Acikel, Mustafa Yilmaz, Metin Karayakali, Kayihan Karaman, Cagri Zorlu, Atac Celik

**Affiliations:** ^1^Department of Cardiology, Gaziosmanpasa University School of Medicine, Tokat, Turkey; ^2^Department of Biochemistry, Gaziosmanpasa University School of Medicine, Tokat, Turkey

**Keywords:** Coronary Artery Disease, Transradial Coronary Angiography, Radial Artery Vasospasm, Adropin, Monocyte Count, Monocyte/HDL Cholesterol Ratio

## Abstract

***Introduction:*** Transradial coronary angiography (TRA) is associated with a lower incidence of bleeding rate and access site complications and is associated with better outcomes compared to transfemoral angiography. However, radial artery spasm (RAS) is an important limitation of TRA procedures. Little is known regarding the relationship of serum vasodilator and inflammatory markers with RAS. Therefore, the present study aimed to investigate the association between serum adropin level and RAS in patients undergoing TRA.

***Methods:*** From February 2020 to January 2021, 39 consecutive patients who underwent elective daiagnostic TRA and experienced RAS during the procedure, and 42 age and sex matched controls who did not experience RAS were prospectively included into the study. The groups were compared regarding serum adropin levels and inflammatory markers.

***Results:*** Although adropin levels were found to be lower in the RAS group, this difference was not statistically significant between the the patients with RAS and controls (14.9 vs. 16.1, *P*=0.105). However, inflammatory parameters monocyte count and MHR (monocyte/HDL cholesterol ratio) were found to be statistically significantly higher in the RAS group compared to controls (*P*=0.001 and *P*=0.010, respectively). Moreover, a significant positive correlation was found between the monocyte count and RAS (r:0.360, *P*<0.001), and between MHR and RAS (r:0.288, *P*=0.009). Furthermore, multivariate analysis demonstrated that monocyte count (OR:1.671, 95%CI:1.312-2.094, *P*=0.001) and MHR (OR:1.116, 95%CI:1.054-1.448, *P*=0.022) were found to be independent predictors of RAS.

***Conclusion:*** Serum vasodilator and inflammatory markers may be useful in the prediction of RAS in patients undergoing TRA procedures.

## Introduction

 Coronary angiography (CAG) is the gold standard imaging method in the diagnosis and treatment of atherosclerotic coronary artery disease (CAD) and transradial coronary angiography (TRA) is associated with a lower incidence of complications, particularly bleeding and access site complications, and seems to be associated with better outcomes compared to transfemoral angiography.^1,2^ However, radial artery spasm (RAS) is an important complication and limitation of TRA procedures, because the radial artery is more prone to spasm development, since it has a large muscular media, smaller calibration, and higher receptor-mediated vascular movement.^3^ Importantly, it has been shown that RAS may be responsible for up to 38% of all TRA procedure failures.^4,5^ RAS generally presents as severe forearm pain combined with difficult manipulation of the catheter or sheath. Clinical predictors of RAS include presence of radial artery anomalies, catheter changes, pain during radial cannulation, using large-diameter catheters, female gender, and small radial artery diameter.^6,7^ However, little is known regarding the relationship of serum vasodilator and inflammatory markers with RAS.

 Adropin is an endogenous polypeptide produced in heart, brain, liver and coronary endothelial cells and increases the secretion of nitric oxide (NO) by increasing the activity of endothelial nitric oxide synthetase (eNOS) and causes vasodilation in the vessel wall with an indirect effect and has a potential endothelial protective role.^8^ Importantly, reduced circulating adropin level is associated with endothelial dysfunction.^9^ Moreover, serum adropin level seems to be decreased in patients with atherosclerotic CAD and coronary slow flow.^9,10^ However, the association of serume adropin level with RAS which is triggered by endothelial dysfunction remains unclear. Therefore, the present study aimed to investigate the association between serum adropin level and RAS in patients undergoing TRA. Additionally, the association of inflammatory markers with RAS was also investigated.

## Materials and Methods

###  Study design

 From February 2020 to January 2021, 39 consecutive patients who underwent elective daiagnostic TRA and experienced RAS during the procedure, and 42 age and sex matched controls who did not experience RAS were prospectively included into the study. Patients with acute coronary syndromes, failed TRA or insufficient visualization of coronary arteries were excluded at baseline. Serum adropin levels were measured for all study participants and groups were compared regarding clinical parameters, inflammatory markers, and serum adropin levels. Demographic characteristics of the patients were recorded at the admission. Biochemical markers were measured from blood samples obtained after 12 hours of fasting. Hypertension was defined as systolic blood pressure ≥140 mmHg and/or diastolic blood pressure ≥90 mmHg, and/or treatment with antihypertensive drugs. Diabetes was defined as at least two fasting blood glucose levels of ≥ 126 mg/dL, or two-hour blood glucose levels of ≥200 mg/dL, or HbA1C level ≥ 6.5, or treatment with antidiabetic medications. Smoking was defined as active smoking.

 The study was conducted in full accordance with the Declaration of Helsinki and approved by the local ethics committee. An informed consent was obtained from all study participants.

###  Adropin level measurement

 Blood samples taken from the patients were centrifuged immediately and the plasmas of the centrifuged blood samples were placed in eppendorf tubes and stored at -80 ˚C until the day of analysis. Plasma samples were gradually thawed and homogenized on the day of the study. The plasmas were processed by keeping them at room temperature on the day of the study. Adropin levels were measured with the FineTest brand Human ENHO (Adropin) ELISA Kit (Wuhan, Fine Biotech Co., Wuhan, Hubei, China) with the ELISA method Sandwich principle. Human ENHO (Adropin) ELISA Kit’s intra-assay CV &lt; 8%, inter-assay CV &lt; It is specified as 10% and its sensitivity is 9.375pg/ml.

###  Transradial angiography

 TRA was performed according to its standard procedure. A 5cc (200 mcg glyceryl trinitrate and 3 cc prilocaine) local anesthesia was applied into the radial artery area. The radial artery was punctured with a 3.8 cm seldinger needle. Subsequently, a 6F radial sheath (7cm) was placed into the radial artery. After the sheath placement, intravenous 5000 units of heparin and 200 mcg of glyceryl trinitrate were routinely administered to all patients. A 0.035 inch hydrophilic or 0.032-inch teflon coated guide wires were used in the procedures to advance the angiography catheters (5F terumo radial catheter or 5F right and left diagnostic/guiding catheters). At the end of the TRA procedure, the sheath was removed, and the radial artery was compressed by the arm cuff. RAS was defined as acute onset pain in the patients’ forearm or arm, and inability to advance the catheter or difficulty in the manipulation of the catheter.

###  Statistical analysis

 Statistical analyses were performed using SPSS software version 22.0. Normality distribution of continuous variables was tested with Kolmogorov-Smirnov or Shapiro-Wilk tests. Descriptive variables were presented using medians and interquartile range for non-normally distributed variables. Since the continuous variables were not normally distributed, the Mann-Whitney U test was conducted to compare these parameters. Categorical data were compared using chi-square test or Fisher’s exact test. Spearman correlation analysis was performed to demonstrate the correlation between RAS and laboratory parameters. Multivariate logistic regression analysis was performed to demonstrate the independent predictors of RAS. A p-value less than 0.05 was considered to indicate statistical significance.

## Results

 The median age of the study participants was 63 years with 59.3% being female. The frequency of hypertension, diabetes and smoking in the study population was 64.2%, 44.4% and 19.8%, respectively. Importantly, there was no statistically significant difference between the patients with RAS and control group regarding cardiovascular risk factors. When comparing the groups regarding adropin levels, although adropin levels were found to be lower in the RAS group, this difference was not statistically significant between the the patients with RAS and controls (14.9 vs. 16.1, p = 0.105). However, in the laboratory analysis, inflammatory parameters monocyte count and MHR (monocyte/HDL cholesterol ratio) were found to be statistically significantly higher in the RAS group compared to controls (p = 0.001 and p = 0.010, respectively). Table 1 demonstrates the clinical and laboratory characteristics of the patient groups. In the gender basis analysis, no statistically significant difference was observed between male and female patients regarding adropin levels (15.9 vs. 15.8, p = 0.475). Importantly, there was a trend toward lower adropin levels in patients with RAS compared to controls in female patients; however, this difference was not statistically significant (14.8 vs. 16.6, p = 0.053). Conversely, adropin levels were found to be numerically higher in patients with RAS compared to controls in male patients; however, this difference was not also statistically significant (16.0 vs 14.4, p = 0.797). Table 2 shows the adropin levesl of female and male patients.

**Table 1:** Clinical and laboratory characteristics of study participants

**Variables**	**All patients** **(n=81)**	**RAS ** **(n=39)**	**No RAS ** **(n=42)**	***P***
Age; median (IQR)	63.0 (55.0; 67.0)	63 (54.5; 66.0)	61.5 (55.0; 67.0)	0.943
Females; n (%)	48 (59.3%)	24 (61.5%)	24 (57.1%)	0.687
Diabetes n (%)	36 (44.4%)	20 (51.3%)	16 (38.1%)	0.233
Hypertension n (%)	52 (64.2%)	27 (69.2%)	25 (59.5%)	0.363
Previous CAD; n (%)	16 (19.8%)	6 (15.4%)	10 (23.8%)	0.341
Smoking; n (%)	16 (19.8%)	8 (20.5%)	8 (19.0%)	0.869
Ad-hoc PCI n (%)	28 (34.6%)	13 (33.3%)	15 (35.7%)	0.822
Adropin (ng/ml) median (IQR)	15.9 (11.9; 18.2)	14.9 (6.6; 17.5)	16.1 (12.1; 18.6)	0.105
Creatinine (mg/dl) median (IQR)	0.79 (0.69; 0.95)	0.80 (0.69; 0.95)	0.77 (0.63; 0.95)	0.640
BMI (kg/m^2^)	29.4 (25.9; 32.1)	30.1 (27.0; 32.8)	27.9 (25.6; 31.2)	0.204
AST(U/L)	18.3 (14.7; 23.0)	18.3 (14.6; 22.3)	18.3 (15.1; 23.1)	0.813
WBC (10³ /mL)	7.2 (6.0; 8.6)	7.8 (6.8; 8.7)	6.9 (6.0; 8.0)	0.070
HGB (gr/dl)	13.1 (12.2; 14.5)	12.8 (12.0; 14.9)	13.3 (12.6; 14.2)	0.561
PLT (10³ /uL)	265 (217;313)	248 (212; 304)	269 (223; 323)	0.182
TSH	1.39 (0.85; 1.99)	1.56 (0.82; 2.09)	1.27 (0.87; 1.89)	0.319
Total Cholesterol (mg/dl)	177 (147; 198)	182 (152; 201)	169 (137; 196)	0.123
LDL-Cholesterol (mg/dl)	120 (91; 142)	124 (103; 141)	112 (81; 140)	0.151
HDL-Cholesterol (mg/dl)	41.2 (36.5; 47.9)	41.4 (35.9; 47.5)	41.1 (36.6; 48.2)	0.777
Triglyceride (mg/dl)	142 (112; 222)	165 (119; 232)	128 (109; 210)	0.183
Neutrophil (10³ /uL)	4.27 (3.53; 5.30)	4.50 (3.95; 5.46)	3.90 (3.17; 5.10)	0.072
Lymphocyte (10³ /uL)	2.12 (1.73; 2.52)	2.02 (1.71; 2.54)	2.13 (1.75; 2.48)	0.839
NLR	1.85 (1.50; 2.78)	2.17 (1.58; 2.84)	1.63 (1.46; 2.49)	0.127
PLR	125 (93; 161)	120 (94; 151)	126 (96; 161)	0.593
Monocytes (10³ /uL)	0.52 (0.40; 0.66)	0.58 (0.45; 0.73)	0.44 (0.34; 0.56)	**0.001**
MHR	12.5 (9.2; 17.4)	13.4 (10.9; 18.1)	10.6 (7.4; 15.4)	**0.010**

Abbreviations: RAS, radial artery spasm; IQR, interquartile range; CAD, coronary arter disease; BMI, body-mass index; AST, aspartate aminotransferase; WBC, white blood cell; HGB, hemoglobin; PLT, Platelet; TSH; thyroid stimulating hormone; LDL-cholesterol, low density lipoprotein cholesterol; HDL-cholesterol, high density lipoprotein cholesterol; NLR, Neutrophil/Lymphocyte ratio; PLR, Platelet/lymphocyte ratio; MHR, Monocytes/high density lipoprotein cholesterol ratio

**Table 2:** Adropin levels of gender subgroups

**Variables**	**Adropin (median, IQR)**	***P***
Males (n=33) Females (n=48)	15.9 (9.3, 18.2) 15.8 (12.4, 18.2)	0.475
**Females (n=48);**		0.053
RAS (n=24)	14.8 (6.8, 17.5)	
No RAS (n=24)	16.6 (14.6, 18.5)	
**Males (n=33);**		0.797
RAS (n=15)	16.0 (8.7, 17.3)	
No RAS (n=18)	14.4 (9.6, 18.6)	

Abbreviations: IQR, interquartile range; RAS, radial artery spasm

 In the correlation analysis, a significant positive correlation was found between the monocyte count and RAS (r:0.360, p<0.001), and between MHR and RAS (r:0.288, p = 0.009). Furthermore, multivariate analysis demonstrated that monocyte count (OR:1.671, 95%CI:1.312-2.094, p = 0.001) and MHR (OR:1.116, 95%CI:1.054-1.448, p = 0.022) were found to be independent predictors of RAS in patients undergoing TRA. Table 3 shows independent predictors of RAS in multivariate analysis

**Table 3:** Independent predictors of radial artery spasm in multivariate analysis

**Variables**	**Odds Ratio**	**95% Confidence interval**	***P***
MHR	1.116	1.054-1.448	**0.022**
Monocyte count	1.671	1.312-2.094	**0.001**

Abbreviations: MHR, Monocytes/ High density lipoprotein cholesterol ratio

## Discussion

 The present study demonstrated that serum adropin level was not associated with RAS occurrence in patients undergoing TRA. However, the relationship of adropin level with RAS was inverse in male and female patients. Moreover, inflammatory markers monocyte count and MHR were found to be significantly associated with RAS emergence, and both parameters were found to be independent predictors of RAS in patients undergoing TRA. To the best of our knowledge, this the the first study that investigated the clinical usefulness of adropin in patients undergoing TRA. Furthermore, our results revaled that inflammatory parameters may be useful in the prediction of RAS in patients undergoing TRA.

 TRA is the recommended method for patients undergoing CAG and is associated with reduced bleeding rates and better outcomes compared to transfemoral angiography.^1,2^ Also, TRA is associated with early mobilization, improved patient comfort, reduced access site and cardiovascular complications and reduced hospital stay.^11^ RAS is an important complication that may occur during TRA procedures. Radial artery is prone to spasm due its small caliber, large muscular media and a higher receptor-mediated vascular movement.^3,12^ Importantly, RAS is more common in women, and its frequency rises when the number of catheters used, and manipulation increases.^6,7^ Moreover, a decrease in the NO levels, which is a vasoactive substance produced from the endothelium and plays an important role in vascular tonus, seems to be associated with RAS occurrence in patients undergoing TRA procedures.^13^ Adropin increases the release of NO by increasing eNOS activity, causes vasodilation in the vessel wall with an indirect effect and has a potential endothelial protective role. Adropin plays a protective role against endothelial dysfunction by regulating eNOS expression through various growth factors and proteins.^14^ Importantly, it has been demonstrated that decreased serum adropin level is significantly associated with CAD.^9,15,16^ Therefore, decreased adropin level may lead endothelial dysfunction that may cause vascular damage and increased vascular tonus. In the present study, there was a trend toward lower adropin levels in patients with RAS compared to controls. However, this difference was not statistically significant. The small number of patients may be the main reason in the lack of statistical significance of this difference. Since the adropin has an endhotelial protective role and causes vasodilation, larger studies may be useful in the defining the association of adropin with RAS emergence in patients undergoing TRA procedures.

 Another important finding of our study is that a significant association of inflammatory markers monocyte count and MHR with RAS was demonstrated. Inflammation plays an important role in the pathophysiology of endothelial dysfunction and atherosclerosis and has a significant effect on vascular tonus.^17^ However, little is known regarding the the relationship of inflammatory markers with RAS in patients undergoing TRA procedures. Monocytes are a source of various cytokines and molecules that interact with endothelial cells and are one of the most important cells in the inflammation. Also, monocytes play an important role in the vascular inflammation and atherosclerosis.^18^ Previous studies have shown that the number of circulating monocytes is increased in people with atherosclerotic disease, and increased monocyte count can independently predict cardiovascular events.^19,20^ Additionally, increased monocyte count seems to be associated with deterioration in the vascular structure.^21^ MHR is also an important inflammatory marker, and it is associated with major cardiovascular adverse events in patients with CAD.^22,23^ MHR seems to be associated with increased oxidative stress and inflammatory status in patients with CAD, and is a predictor of severe and complex coronary atherosclerosis.^24,25^ Also, increased MHR is a sign of endothelial dysfunction, and is significantly associated with abnormal vascular structure and functions.^26-28^Therefore, current evidence suggest that monocyte count and MHR seem to be associated with inflammation and endothelial dysfunction in patients with CAD. Our results revealed that both inflammatory markers were independently associated with RAS emergence and may be useful in clinical practice to distinguish the high-risk patients for RAS in patients undergoing TRA procedures.

 The present study has some limitations. First, low sample volume and study power is the major limitation. However, it should be considered that the study was conducted during the covid-19 pandemic. Larger studies are necessary to demonstrate the association between adropin level and RAS. Second, since the radial artery angiography was not routinely performed during TRA procedures, radial artery diameter could not be measured. In this study, RAS was defined according to clinical findings and new onset symptoms, like previous studies.

## Conclusion

 The present study demonstrated that serum adropin level was found to be lower in patients experienced RAS compared to the controls, however, this difference was not statistically significant. The relationship of adropin with RAS needs to be investigated in larger studies. Moreover, a significant association between inflammatory markers and RAS was found. Monocyte count and MHR were found to be independent predictors of RAS in patients undergoing TRA procedures. Since the endothelial dysfunction and inflammation seem to be associated with occurrence of RAS, endothelial biomarkers and inflammatory parameters may be useful in clincal practice to define the patients at high-risk for emergence of RAS during TRA procedures.

## Funding

 None.

## Ethical approval

 This study was approved by Tokat Gaziosmanpasa University Clinical Research Ethics Committee on 06 February 2020 with project number 20-KAEK-012.

## Competing interest

 The authors declare that they have no conflicts of interest.
